# Downhill Running: What Are The Effects and How Can We Adapt? A Narrative Review

**DOI:** 10.1007/s40279-020-01355-z

**Published:** 2020-10-09

**Authors:** Bastien Bontemps, Fabrice Vercruyssen, Mathieu Gruet, Julien Louis

**Affiliations:** 1 Université de Toulon, Laboratoire IAPS, Toulon, France; 2 Research Institute for Sport and Exercise Sciences, Liverpool John Moores University, Liverpool, L3 3AF UK

## Abstract

Downhill running (DR) is a whole-body exercise model that is used to investigate the physiological consequences of eccentric muscle actions and/or exercise-induced muscle damage (EIMD). In a sporting context, DR sections can be part of running disciplines (off-road and road running) and can accentuate EIMD, leading to a reduction in performance. The purpose of this narrative review is to: (1) better inform on the acute and delayed physiological effects of DR; (2) identify and discuss, using a comprehensive approach, the DR characteristics that affect the physiological responses to DR and their potential interactions; (3) provide the current state of evidence on preventive and in-situ strategies to better adapt to DR. Key findings of this review show that DR may have an impact on exercise performance by altering muscle structure and function due to EIMD. In the majority of studies, EIMD are assessed through isometric maximal voluntary contraction, blood creatine kinase and delayed onset muscle soreness, with DR characteristics (slope, exercise duration, and running speed) acting as the main influencing factors. In previous studies, the median (25th percentile, *Q*_1_; 75th percentile, *Q*_3_) slope, exercise duration, and running speed were − 12% (− 15%; − 10%), 40 min (30 min; 45 min) and 11.3 km h^−1^ (9.8 km h^−1^; 12.9 km h^−1^), respectively. Regardless of DR characteristics, people the least accustomed to DR generally experienced the most EIMD. There is growing evidence to suggest that preventive strategies that consist of prior exposure to DR are the most effective to better tolerate DR. The effectiveness of in-situ strategies such as lower limb compression garments and specific footwear remains to be confirmed. Our review finally highlights important discrepancies between studies in the assessment of EIMD, DR protocols and populations, which prevent drawing firm conclusions on factors that most influence the response to DR, and adaptive strategies to DR.

## Key Points

**Table:** 

Due to its eccentric nature, downhill running (DR) induces lower limb muscle damage, manifested by alterations in muscle structure, muscle function, and ensuing running performance for up to several days after exercise.
Manipulating DR characteristics (slope, running speed, and duration), independently or not, can influence the extent of exercise-induced muscle damage (EIMD). Although trained and/or accustomed people generally experience less muscle damage following DR, it is still unknown if sex and/or age may influence the adaptation to DR.
Scientific evidence suggests preventive strategies that consist of prior exposure to DR to limit the extent of muscle damage induced by DR.
Evidence is lacking to support the use of in-situ strategies such as compression garments, specific footwear, or modification in running stride to limit muscle damage induced by DR, which highlights the need for further high-quality research.

## Introduction

Eccentric muscle contractions occur when the magnitude of the force applied to the muscle exceeds the strength produced by the muscle itself (i.e. negative work), resulting in a lengthening action of the musculotendinous system. Eccentric muscle contractions are known to induce high mechanical strain on the musculotendinous system, leading to moderate to severe exercise-induced muscle damage (EIMD) [1].

In laboratory settings, local (i.e. single muscle group tested with an isokinetic dynamometer) and whole-body eccentric-based models such as eccentric cycling and downhill running (DR) have been used to examine the physiological consequences of eccentric muscle actions and/or EIMD [2]. For example, intense and/or prolonged DR is well-known to induce muscle functional alterations [3]. More precisely, DR generally leads to substantial neuromuscular fatigue which can originate from both peripheral and central mechanisms. The peripheral component of neuromuscular fatigue may be attributed to longer muscle length (i.e. overstretched sarcomeres) during eccentric muscle actions over braking phases, leading to myofibrillar damage such as disrupted weaker sarcomeres and/or excitation–contraction coupling failure [1, 4]. The central component of neuromuscular fatigue may be attributed to spinal and/or supraspinal factors [5, 6]. Thus, the DR modality has often been used for different purposes including: (1) studying the recovery kinetics of physical performance following muscle damage (e.g. [7–9]); (2) testing the reliability and validity of varied techniques to assess EIMD (e.g. [6, 10]); (3) studying the effects of strenuous exercise on physiological adaptations and/or alterations (e.g. [11–13]); (4) testing different strategies to attenuate EIMD and/or improve subsequent recovery (e.g. [14–16]).

In a sporting context, DR sections can be part of off-road running races mostly taking place in natural environment and unsealed roads (e.g. trail running, mountain running, fell running and cross-country running, orienteering, obstacle course racing or ultramarathon running, [17]) or road running races including downhill sections (e.g. the St. George marathon, St George, USA; the Holualoa Tuscon marathon, Tuscon, USA; the Big Sur International marathon, Big Sur, USA) [18, 19]. Due to its eccentric-based muscle action modality, DR may play a major role in the occurrence of neuromuscular fatigue (especially due to peripheral factors) and muscle damage, and thus represents a main challenge for runners. For example, the maximal strength production capacity of lower-limb muscles (i.e. knee extensors, KE, and plantar flexors, PF), often assessed through isometric maximal voluntary contraction (MVC) is generally dramatically reduced (− 15 to − 40%) following trail running races (i.e. the most popular off-road running races, taking place in natural environment over a variety of terrains with minimal paved or asphalt roads; for review, [17]) (e.g. [19–22]). Although DR characteristics (running speed and/or intensity, running duration and slope) vary among running disciplines, they do not predispose to the extent of an alteration in strength production capacity [3, 23]. For example, comparable force/torque losses and peripheral alterations were reported following a 6.5 km DR at maximal speed (altitude drop: 1264 m; average slope: − 16.8%) and in ultra-marathons (i.e. > 42.195 km) in experienced trail runners (for review, [3]). In line with these observations, it has been suggested that performance in trail running is largely influenced by the demanding nature of graded sections (especially DR sections where eccentric muscle actions are predominant), leading to EIMD including neuromuscular fatigue [3]. The same hypothesis may be applied to other running disciplines that include notable DR sections.

On the other hand, DR could be used during specific training periods to improve running performance via neural and musculotendinous adaptations. Some narrative reviews have focused on the physiological and/or biomechanical adaptations to trail running and/or prolonged graded running [18, 23]. However, a comprehensive analysis of the effects of DR on both in-situ physiological responses and recovery kinetics (i.e. acute and delayed physiological responses and EIMD markers) according to training level is lacking. In parallel, anecdotal data from field observations and a growing number of research studies show an interest in strategies to facilitate the adaptation to DR, namely reduce EIMD. These strategies include for example, DR training, wearing compression garments and using specific footwear, though it is difficult to draw conclusions due to the variety of results.

Within this framework, the purpose of this narrative review is threefold: (1) to further our understanding of the physiological responses to DR; (2) to identify and discuss, using a comprehensive approach, the DR characteristics that affect the physiological responses to DR and their potential interactions; (3) to provide a synthesis of evidence on strategies to better tolerate DR by limiting EIMD. Manuscripts were acquired by searching the electronic databases of the US National Library of Medicine (PubMed), ScienceDirect and SPORTDiscus using the following keywords: “running” AND “downhill” OR “decline” OR “grade” OR “gradient” OR “slope” OR “muscle damage” OR “muscle fatigue” OR “biomechanics” OR “strategy” OR “compression garment” OR “running garment” OR “preconditioning” OR “training” OR “repeated bout effect” OR “footwear”. Electronic database searching was supplemented by examining reference sections of relevant articles (i.e. hand search). The literature search was conducted up to March 2020. In the entire manuscript, we limited our analysis to studies including DR only as a modality of whole-body eccentric-biased exercise, with human participants only. We did not consider studies using other means (e.g. steps, one-legged DR, sprint-assisted DR, eccentric cycling or eccentric muscle actions on isokinetic dynamometer) of generating muscle damage or eccentric muscle actions. In studies including DR exercises that also tested a nutritional strategy or a strategy to limit EIMD, only the placebo groups (with no strategy) were included in the analyses performed in the second part of the manuscript to identify and discuss DR factors responsible for EIMD and their interaction. No limit to the search domain was applied regarding population characteristics, training level, and DR characteristics (i.e. running speed and/or intensity, exercise duration, and slope). In the present review, the generic term ‘EIMD” refers to direct markers of muscle damage including ultrastructural alterations (e.g. myofibrillar disruption) as well as indirect markers such as physiological (e.g. systemic efflux of myocellular enzymes and proteins), perceptual (e.g. delayed onset muscle soreness) and functional alterations (e.g. loss in MVC force/torque) following DR [24]. Functional alterations refer to the concept of “neuromuscular fatigue” defined as any exercise-induced reduction in voluntary maximal force or power [25].

## Muscular Alterations Following Downhill Running

Grade-specific biomechanical and neuromuscular alterations occur in running (for review, [18]). When compared to level running, DR is characterized by repeated and prolonged eccentric muscle actions of lower-limb muscles [14, 24], lowered capacity of the stretch–shortening cycle [26, 27], alterations in foot strike pattern (usually rear-foot strike pattern, i.e. heel contacts first) [27–29], changes in ground reaction forces (larger normal impact force and greater parallel braking force compared to level running, whatever the slope studied) [27, 30], specific mechanical energy fluctuations of the centre of mass during each stride (e.g. greater negative external work and lower positive external work, i.e. the work done to decelerate and accelerate the body’s centre of mass with respect to the environment, respectively) [26, 29, 31–34], and alterations in impact shock attenuation (e.g. greater tibial acceleration) [27, 33, 35–37]. Those grade-specific alterations originate from the greater mechanical strain applied to the lower-limb musculoskeletal system during the foot–ground contact time in DR, which may lead to accentuated acute and delayed EIMD (e.g. structural muscle alterations, leakage of specific-muscle proteins into the bloodstream, reduction in MVC force/torque) [3, 38], and consequently alteration in running economy (RE) and performance [3, 18]. As such, a better understanding of these alterations following DR is the first stage in our aim to investigate the DR characteristics responsible for such alterations, and adaptative strategies.

### Structural Muscular Alterations Following Downhill Running

The presence of EIMD following strenuous exercise can be assessed through histological analysis of biopsy samples and magnetic resonance imaging (MRI) of muscle groups (KE and PF are the muscle groups the most investigated) involved in DR. Myofibrillar disruptions and necrosis in lower-limb (e.g. *vastus lateralis*) muscles are generally reported after eccentric exercise [39]. Following 30-min treadmill DR (slope: − 12%; intensity: 80% of maximal aerobic velocity recorded on level grade), Féasson et al. [40] reported important ultrastructural muscular alterations in the *vastus lateralis* of healthy males. These alterations included streaming, disruption and dissolution of Z-disk structures associated with A-band disruption and misalignment of myofibrils, and disturbances of the cross-striated band pattern along the longitudinal sections. Increases in skeletal muscle mRNA expression of several inflammation-related genes, increases in pro-inflammatory cytokine concentration, and satellite cells proliferation in KE muscles were also reported after DR [41, 42]. Using MRI, Maéo et al. [43] also reported structural muscular alterations (i.e. local inflammatory oedema estimated via transverse relaxation time, *T*_2_) in both KE and PF muscle groups following 45-min treadmill DR (slope: − 15%; average speed: 10.0 km h^−1^) in healthy young adults.

The important leakage of muscle-specific proteins (creatine kinase, CK; myoglobin, Mb; and lactate dehydrogenase, LDH) into the blood, accompanied by increases in muscle Ca^2+^ and K^+^ content following strenuous exercise, are also considered common indirect markers of EIMD [44–47]. Two main mechanisms could explain this efflux of muscle-specific proteins into the blood stream. Firstly, muscle fibres may exhibit an increased membrane permeability following an increase in cytoplasmatic Ca^2+^ through an inhibition of the Ca^2+^ pump that actively transports Ca^2+^ out of the cell, thus promoting the activation of the K^+^ channel and leading to membrane damage [48]. In addition, this could be explained by local tissue damage induced by the eccentric muscle actions which may cause a degeneration of the muscle structure [49]. Peake et al. [50] reported a large increase (+ 1800%) in plasma Mb concentration 1-h post-DR (45 min at 60% $\dot{V}{\text{O}}_{{{\text{2max}}}}$, − 10% slope; with $\dot{V}{\text{O}}_{{{\text{2max}}}}$ corresponding to the maximal aerobic capacity) followed by a large increase (+ 420%) in plasma CK concentration 24-h post-DR in well-trained runners. This efflux of muscle-specific proteins into the blood stream may persist for several days following DR. For example, Malm et al. [38] reported a significant efflux of serum CK (+ 150%) up to 7 days following DR.

These structural muscular alterations occurring during DR may be accompanied by MVC force/torque decrements [51, 52] and thus, reflect the level of neuromuscular fatigue [25]. Since the manifestation of MVC force/torque loss and structural muscular alteration are concomitant [53, 54], it appears important to discuss their relationship.

### Functional Muscular Alterations Following Downhill Running

Loss of MVC force/torque is commonly used to assess the global state of neuromuscular fatigue that may originate from both peripheral and central levels [25]. Because the extent of force/torque loss is also influenced by the number of muscle fibres suffering from myofibrillar disruption and/or excitation–contraction failure [49, 53, 55–57], it may provide information on the extent of EIMD [54]. The reduction in MVC force/torque may depend on the exercise modality (i.e. grade), exercise characteristics (duration, running speed and/or intensity) and population characteristics (age, sex, training level). For example, Garnier et al. [58] reported greater decrements in isokinetic (concentric and eccentric) MVC torque of KE, accompanied by a greater occurrence of the peripheral component of neuromuscular fatigue, in-situ perceived exertion (in *gluteus* muscles) and delayed muscle pain (in KE, PF and *gluteus*) following a 45-min DR bout, compared to uphill and level running bouts performed at the same relative intensity (same reserve heart rate, HR).

Immediately after DR, large reductions in isometric MVC force/torque of KE and PF muscles are generally reported, and may vary from − 14 to − 55% for KE [5, 9, 10, 15, 38, 43, 58–87] and from − 15 to − 25% for PF [9, 15, 43]. Similarly, a decline in muscle performance (expressed as MVC force/torque loss or endurance capacity) was reported during isokinetic muscle actions (tested from 0.52 to 5.2 rad s^−1^ and from 0.52 to 2.6 rad s^−1^ for concentric and eccentric modalities, respectively) [58, 88, 89], dynamic strength measurement such as counter movement jump [15, 90] and muscle endurance test [91]. Decrements in MVC force/torque generally last up to 4–5 days before complete recovery [38, 70, 73, 92].

DR is well-known to alter muscle structure and to induce neuromuscular fatigue. More specifically, the acute reduction in MVC force/torque following DR is typically associated with a reduction in maximal central drive (i.e. a mechanism associated with a central component of neuromuscular fatigue, assessed via maximal voluntary activation, VA; from − 2.5 to − 8% in KE and PF; [9, 10, 15]) and the so-called “low-frequency fatigue”. The latter (usually investigated via muscle or nerve electrical stimulation) manifests through a decrease of the low (10–20 Hz)-to-high (50–100 Hz) frequency doublet ratio [93], reflecting a reduction in Ca^2+^ release from the sarcoplasmic reticulum [93]. It is thus likely that the failure in excitation–contraction coupling is mostly responsible for the occurrence of the peripheral component of neuromuscular fatigue following DR. In addition, the acute reduction in maximal central drive immediately after DR may contribute to the acute reduction in MVC force/torque. Such reduction in VA may originate from the spinal and/or supraspinal level, with for instance a role of reflexes mediated by muscle III-IV afferents that may reduce the recruitment and/or firing rates of motoneurons [25]. In previous studies, the reduction in maximal central drive was usually observed through peripheral nerve stimulation (PNS). However, PNS does not allow quantification of the source of drive to the motoneurons making unclear the relative contribution of spinal and supraspinal components in the reduction of MVC force/torque. Bulbulian and Bowles [7] investigated the spinal component using the Hoffman reflex (H-reflex) expressed as a ratio of the maximal electrically stimulated muscle action potential (*H*_max_/*M*_max_ ratio). The authors reported a reduction in *H*_max_/*M*_max_ ratio following a 20-min DR (slope: − 10%; intensity: 50% $\dot{V}{\text{O}}_{{{\text{2max}}}}$) reflecting a reduction in motoneuron pool excitability, in higher proportion compared to a level bout (e.g. − 24.6% versus − 9.3%, respectively) performed at a comparable metabolic cost (expressed as %$\dot{V}{\text{O}}_{{{\text{2max}}}}$). It is possible that repeated and/or prolonged eccentric muscle actions could potentially damage muscle spindles [94, 95], which may impair motoneuron excitability by reducing I_a_ afferents input to the motoneuron pool. However, the H-reflex does not allow inferences on the potential role of supraspinal mechanisms implicated in the reduction of MVC force/torque post DR. A recent study reported an increased corticospinal excitability (assessed through motor-evoked potentials elicited by transcranial magnetic stimulation and recorded from the *abductor pollicis brevis*) 30-min post DR (duration: 30 min; slope: − 10%; intensity: 75% HR_reserve_). However, the absence of cortical VA measurements in this study makes unclear the potential role of changes in corticospinal excitability/inhibition in the occurrence of supraspinal fatigue post DR. Future studies should combine measures of corticospinal excitability and inhibition (e.g. using paired-pulse transcranial magnetic stimulation) with measures of cortical VA, obtained from muscles directly mobilised during DR (e.g. KE and/or PF). Furthermore, although no comparison analysis of VA was conducted between laboratory and ecological settings, the DR surface could influence the magnitude of acute and delayed central fatigue. A greater reduction in VA could be expected upon completion of an ecological DR session, due to the higher cognitive demands of running on technical single tracks, compared to laboratory conditions. For instance, DR in an ecological setting, compared to a laboratory, may further tax cognitive processes such as working memory and spatial navigation planning and thus exacerbate the activation of cortical areas such as the prefrontal cortex (PFC) [96]. A greater mobilization of brain resources may be expected during DR in an ecological setting, with the necessity to accomplish the motor task while dealing with the additional cognitive demand (i.e. dual task). For example, a greater and earlier depletion of brain resources in cortical structures such as the PFC may negatively impact the activity of interrelated motor areas such as the motor cortex, and lead to a greater reduction of VA. In line with this assumption, Mehta and Parasuraman [97] reported a decrease in PFC oxygenation at exhaustion following a fatiguing cognitive-motor dual task (i.e. submaximal handgrip and concomitant mental-math task) compared to the motor task performed alone. Moreover, Chatain et al. [98] found a greater decrease in VA during a fatiguing cognitive-motor dual task (i.e. intermittent isometric quadriceps contractions with concomitant n-back task) compared to the motor task alone. Because replicating the physiological and cognitive demands of ecological DR in a laboratory setting is challenging, this paradigm may be tested during treadmill DR by adding a concomitant cognitive task that may replicate the demanding real-life environment characterizing off-road running (e.g. using virtual reality technology).

**Fig. 1:**
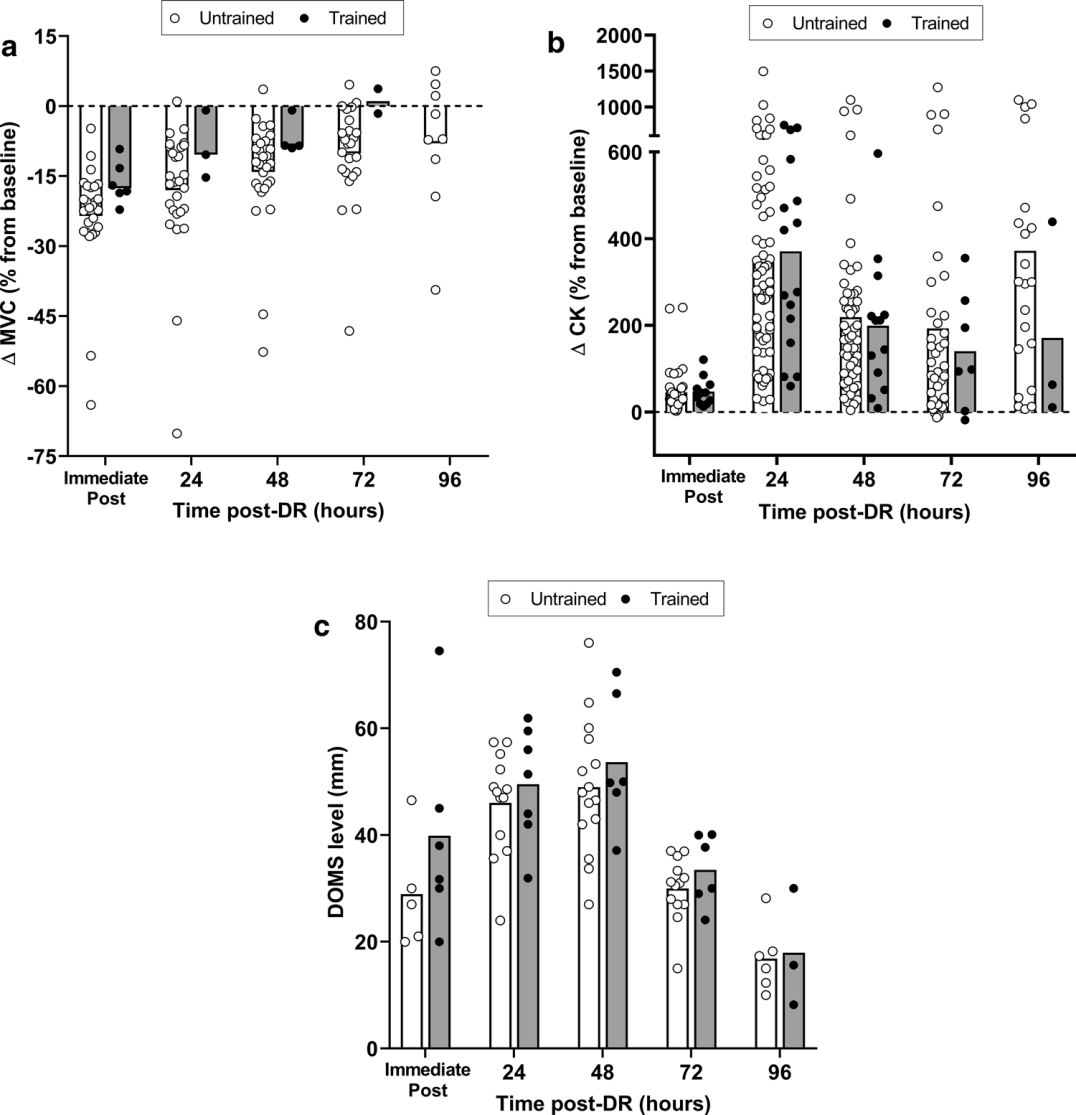
**a** Decrease (% from baseline) in isometric maximal voluntary contraction (MVC) torque of knee extensors after downhill running (DR); **b** increase (% from baseline) in blood (plasma and serum) creatine kinase (CK) concentration after DR; **c** delayed onset muscle soreness (DOMS) response (0–100 mm evaluated on visual analogue scale) for knee extensors after DR. Based on data from original research articles reporting isometric MVC force/torque decrements (*n* = 37; [5, 9, 10, 15, 38, 43, 58–87]), blood CK elevation (*n* = 83; [14, 16, 38, 40–42, 44, 46, 50, 59–62, 65–67, 70, 72–75, 77, 80, 81, 84, 85, 87–90, 92, 121, 134–183]) and DOMS (*n* = 23; [8, 15, 38, 58, 60, 66, 71, 75, 84, 89, 135, 151, 156, 159, 171, 178, 179, 182, 184–188]) responses immediately post-, 24 h post-, 48 h post-, 72 h post- and 96 h post-DR. In all panels, circles and bars refer to individual and mean data, respectively, in white and black for untrained (i.e. healthy and/or recreationally active) and trained populations (i.e. $\dot{V}{\text{O}}_{{{\text{2max}}}}$ > 54–60 ml min kg^−1^ and were involved in endurance-based activities at least 3 times/week), respectively. In the cases where data were not fully presented in the manuscript, data were extracted from original figures using ImageJ software (ImageJ V.1.45 s, National Institute of Health, MD, USA)

It is well-reported that MVC force/torque is impaired for several days following DR (up to 4–5 days for a complete recovery; see Fig. 1). However, a better understanding of the mechanisms responsible for delayed MVC force/torque loss is warranted. Firstly, it is likely that several mechanisms related to EIMD could play a major role in delayed MVC force/torque loss [99, 100], including structural muscular alterations (e.g. sarcomeres ‘popping’-disruption, streaming, disruption, and dissolution of Z-disk structures) and impairments in the excitation–contraction coupling (e.g. damaged T-tubules, sarcoplasmic reticulum and sarcolemma, impaired Ca^2+^ release by the sarcoplasmic reticulum and impaired myofibrillar sensibility to Ca^2+^). To date, only a few studies have investigated recovery (expressed as MVC torque and coupled with peripheral and central assessments of neuromuscular fatigue) up to 48-h post DR [9, 15]. Although Ehrström et al. [15] reported a low-frequency force depression 24-h post DR (duration: 40 min; slope: − 15%; intensity: 55% $\dot{V}{\text{O}}_{{{\text{2max}}}}$), such alterations were not recorded 48-h after a 6.5-km ecological DR [9] where isometric MVC torque was still reduced. It is possible that the prolonged low-frequency force depression may be underestimated within days following DR, as it has been observed after an isokinetic eccentric exercise (200 eccentric MVCs of dorsiflexor muscles) [101]. The decrement in low-frequency force could be higher and/or longer than the aforementioned results and thus may partly explain the prolonged MVC force/torque loss. Moreover, a modification in the muscle length-tension relationship up to 48-h post could partly explain the MVC force/torque decrement following DR, as observed following eccentric-biased exercises [102, 103]. Finally, the important leakage of CK, Mb and LDH into the bloodstream, often associated with the appearance of delayed onset muscle soreness (DOMS) [53] and the inflammatory response, may contribute to delayed MVC force/torque loss [104]. Although the relationship between the inflammatory response, DOMS and MVC torque is not clear [105], all these alterations tend to disappear within 4–10 days post DR [71, 73]. It is noteworthy that few studies have compared the extent of neuromuscular fatigue between KE and PF following DR, with contrasting results [9, 15, 43]. Regarding isometric MVC torque, some studies showed greater decrements in KE than in PF [15, 43] whereas Giandolini et al. [9] reported the contrary. The latter results appear difficult to interpret, since higher peripheral alterations (e.g. amplitude torque reduction to evoked twitch torque, M-wave and 10 Hz-to-100 Hz ratio) were reported in KE despite lower isometric MVC torque loss after DR. These conflicting results warrant the need for additional studies investigating possible muscle-specific adaptations to DR.

While DR studies have mostly investigated the alteration of muscle function using isometric testing, this may not represent the most relevant testing modality. Indeed, single-joint sustained maximal contractions do not represent the real demand of most activities [106]. Future studies should combine various approaches including assessment of isometric and eccentric (recommended from 0.52 to 2.1 rad s^−1^) MVC force/torque, and whole-body (e.g. counter movement jump or squat jump) or isolated power/maximal velocity tests to better characterize EIMD.

### Running Economy Alterations Following Downhill Running

In contrast with the aforementioned EIMD and muscle fatigue variables, RE may be measured during exercise and not only in a “pre-post” experimental design. RE is considered a major determinant of running performance [107, 108] and success in competitive distance running [109, 110]. It is typically measured to evaluate any form of intervention on global metabolic efficiency (e.g. [73, 92, 111]). In DR studies, RE is traditionally defined as the submaximal and steady-state oxygen uptake ($\dot{V}{\text{O}}_{{2}}$, mlO_2_ min^−1^ kg^−1^ with rate of exchange ratio, RER < 1) for a given running speed and is generally assessed on level grade (e.g. [73, 92, 112]). However, RE is often used synonymously with other indicators of metabolic efficiency. In such cases, RE may be expressed as oxygen cost (mlO_2_ kg^−1^ km^−1^) from the body mass-specific $\dot{V}{\text{O}}_{{2}}$. Given that substrate used to cover energy expenditure may vary according to exercise intensity and duration (for review, [113]), interpretations resulting only from the use of milliliters of oxygen (including oxygen cost) must be made with caution. For these reasons, expressing RE as gross energy cost (in J kg^−1^ m^−1^ or kcal kg^−1^ km^−1^), where each $\dot{V}{\text{O}}_{{2}}$ valued is converted to its metabolic energy equivalent (which depends on the RER) would be more appropriate [114]. In the literature, an enhanced RE is associated with a lower oxygen demand, energy cost or oxygen cost for a given running speed.

The negative slope of the terrain could also influence RE due to the higher proportion of eccentric muscle actions compared to flat running. Although the recruitment order of motor units has been recently reported to be similar during submaximal shortening and lengthening contractions, the discharge rate is systematically lower during lengthening actions (for review, [115]) and thus, might lower the metabolic demand [116]. DR would be metabolically less demanding than level running and uphill running for a similar running speed and/or relative intensity (e.g. mechanical power, HR and $\dot{V}{\text{O}}_{{2}}$) [28, 31, 117–120]. For instance, at 75% HR_reserve_, Garnier et al. [117] linked the lower alteration in RE (expressed as $\dot{V}{\text{O}}_{{2}}$) during DR to a greater involvement of passive elastic structures in strength production compared to level and uphill treadmill exercise. The optimum slope (for which the metabolic demand is the lowest) is still a matter of debate due to the variety in DR protocols used and training levels of study participants.

Cardiorespiratory changes (e.g. increase in HR, $\dot{V}{\text{O}}_{{2}}$, and minute ventilation) for a set speed [117, 121, 122], accompanied by an increase in core body temperature [121], generally occur during intense and/or prolonged DR sessions, and may be dependent on the slope that is used [123]. For example, a ~ 10% increase in $\dot{V}{\text{O}}_{{2}}$ was measured immediately after a prolonged DR (30–40 min) in recreational runners [121, 122] whereas the increase was lower (+ 6.8%) in well-trained runners [15]. A reduced amplitude of the $\dot{V}{\text{O}}_{{2}}$ slow component was also observed during a 15-min DR bout (slope: − 15%; average speed: 8.5 km h^−1^) in well-trained runners following the completion of a 15-min uphill bout (at the same running speed, with 10-min recovery between bouts [124]).

Alterations in RE were reported in various prolonged DR protocols and within several hours to days following DR [71, 73, 84, 112, 121, 122]. A 3–18% decrease in RE (expressed as $\dot{V}{\text{O}}_{{2}}$ or energy cost) was reported during flat treadmill running sessions performed immediately after DR [71, 73, 84, 112] and may be associated with an increase in stride frequency and/or reduction in the range of motion of lower-limb joints [64, 71, 73, 84, 92, 112]. Chen et al. [71] reported a 4–7% increase in $\dot{V}{\text{O}}_{{2}}$ up to 3 days after a 30-min DR (slope: − 15%; intensity: 70% $\dot{V}{\text{O}}_{{{\text{2max}}}}$), regardless of running speed [73]. While the time-course of RE alterations seems similar between studies, it was suggested that participants unaccustomed to running and/or untrained in running could experience greater alterations in RE during, immediately after and within days following DR [84].

The change in discharge rate of motor units over time [115], the preponderant type II muscle fiber recruitment [1, 122], and substantial normal impact force and parallel braking force [27] could account for the increased metabolic demand at the end of prolonged DR. This is aligned with the fact that type II fibres, which are energetically less efficient than type I fibres [125], may be further recruited following DR to maintain force production [126], whereas type I fibres could preferentially be recruited during DR [122]. RE alteration after DR may depend on the extent of EIMD, since a greater neural input to the muscle is required to offset the lack of efficiency of damaged fibres [122, 127], and to maintain the force production capacity (particularly necessary during the push-off phase of the running cycle) [128]. In contrast, Lima et al. [84] have recently demonstrated that alterations of RE (expressed as $\dot{V}{\text{O}}_{{2}}$) following DR (up to 3 days after one 30-min DR bout; slope: − 15%; average speed: 9.9 km h^−1^) were not explained by changes in markers of EIMD (serum CK elevation, DOMS, changes in muscle function) or alterations in running mechanics. The authors speculated that other parameters might alter RE following DR, including haemodynamic alterations (such as damaged capillaries [129], compromised flow-mediated vasodilation [130] and oxygen delivery [131] to the damaged sites) and the increase in resting energy expenditure [132].

Taken together, these data provide evidence that prolonged DR can lead to substantial physiological and biomechanical alterations, altering RE and subsequent running performance. While EIMD is well-known to be the main disruptor of running performance and subsequent recovery, underlying mechanisms which could influence the magnitude of muscle damage following DR must be clarified.

## Factors Influencing The Magnitude of Muscle Damage Following Downhill Running

Although the physiological responses to DR have been extensively investigated, the influence of DR characteristics (i.e. slope, running speed and/or intensity, and exercise duration) on the magnitude of EIMD is still unclear. The variety in population characteristics (i.e. training status, sex, age) among studies also adds complexity to our understanding. As discussed in the previous section, muscle damage following DR is classically monitored within the first 24-h to 96-h following exercise via indirect markers of muscle structure, muscle function and perceptions of muscle soreness. As such, we analysed the time-course of changes in isometric MVC force/torque (for KE only), DOMS (for KE only using a 0-100 mm visual analogue scale) and blood CK, the most reported markers in the literature, within this time window. The purpose of this comprehensive analysis is to present the current state of knowledge on how DR characteristics influence muscle damage post DR while highlighting the lack of data in certain areas. The main factors included in the analysis were the training level (trained *vs* untrained in running) and DR characteristics (exercise duration, running speed and slope), while additional factors (age, sex) were also discussed.

### Influence of Training Level and Downhill Running Characteristics

To investigate the effect of training level on indirect markers of EIMD, data extracted from original articles were separated into two different groups: untrained and trained populations. Untrained populations are described as healthy and/or recreationally active. In contrast, trained populations generally present a $\dot{V}{\text{O}}_{{{\text{2max}}}}$ > 54–60 ml min kg^−1^ and are involved in endurance-based activities (i.e. long-distance running, triathlon, trail running) at least 3 times/week [133].

The literature describes acute and delayed changes in indirect markers of muscle damage after DR (Fig. 1). The reduction in isometric MVC force/torque generally peaks immediately post-DR [5, 9, 10, 15, 38, 43, 58–87], whereas increases in blood CK [14, 16, 38, 40–42, 44, 46, 50, 59–62, 65–67, 70, 72–75, 77, 80, 81, 84, 85, 87–90, 92, 121, 134–183] and DOMS [8, 15, 38, 58, 60, 66, 71, 75, 84, 89, 135, 151, 156, 159, 171, 178, 179, 182, 184–188] peak from 24 to 48 h post-DR. Immediately post DR, a greater reduction in isometric MVC force/torque is observed in untrained (mean: − 23.5%; 95% CI: [− 26.9% to − 17.2%] compared to trained populations (− 16.4%; 95% CI: [ − 19.5% to – 12.3%]). In addition, the recovery of isometric MVC force/torque seems to be faster in trained compared to untrained populations (return to baseline 72 h post *vs.* no full recovery 96 h post, respectively). The peak of DOMS is identified 48 h after DR and seems to be of similar magnitude between populations (49.0 mm; 95% CI: [42.0–58.0 mm] and 53.7 mm; 95% CI: [45.3–67.50 mm] in untrained and trained participants, respectively). Finally, the peak increase in blood CK is identified 24 h post DR in similar proportion between populations (+ 346.5%; 95% CI: [+ 164.7% to + 495.5%] and + 370.7%; 95% CI: [+ 173.6% to + 559.1%] in untrained and trained participants, respectively). However, it is possible that the leakage into the bloodstream is less pronounced 72 h and 96 h post DR in trained compared with untrained populations.

Endurance training seems to limit the occurrence of muscle damage following DR [3, 15]. Physical training has been shown to promote higher physiological muscle resistance to exercise due to a combination of neural (motor unit recruitment), morphological (muscle–tendon unit stiffness) and structural adaptations (muscle fascicle pennation angle, and fascicle length) [189, 190]. These data support the importance of training status and training modality to reduce the occurrence of EIMD. It is important to note that, in most research studies, endurance trained populations were not familiarised with DR and/or eccentric-based exercises. A greater training effect can be expected in participants familiarised with DR. They could benefit the most from specific training adaptations due to the important eccentric component of DR and, thus, limit the magnitude of muscle damage following DR.

**Fig. 2:**
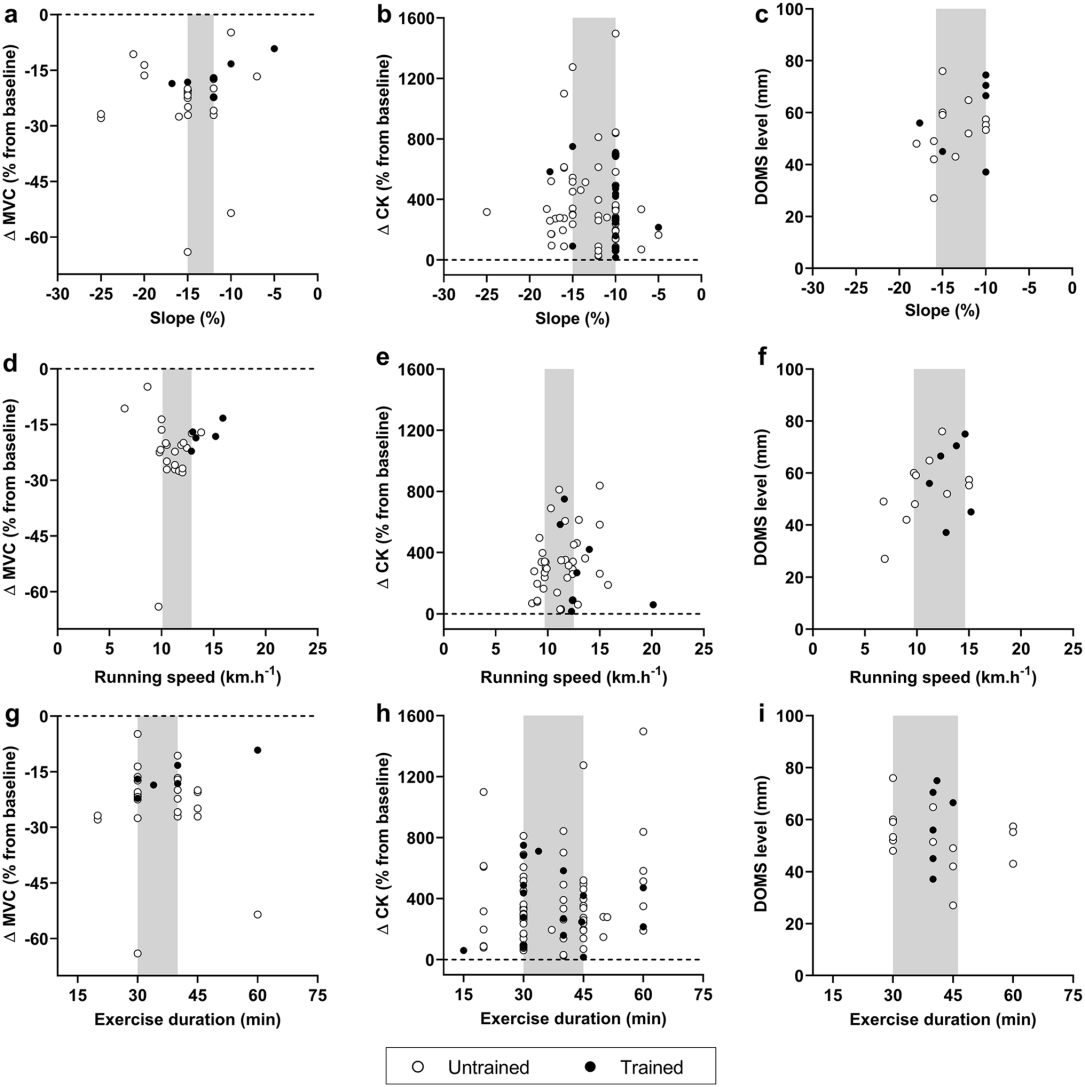
Relationships between peak changes in indirect markers of exercise-induced muscle damage (i.e. isometric maximal voluntary contraction, MVC; blood (plasma and serum) creatine kinase, CK; and delayed onset muscle soreness, DOMS) and downhill running (DR) characteristics (i.e. slope (**a**–**c**), running speed (**d**–**f**) and exercise duration (**g**–**i**)]. All data presented and DR characteristics were extracted from original articles. Peak MVC force/torque decrement was reported immediately post-DR. Peak changes in blood CK and DOMS were reported between 24-h post and 48-h post DR. White and black circles refer to original data from untrained (i.e. healthy and/or recreationally active) and trained populations (i.e. $\dot{V}{\text{O}}_{{{\text{2max}}}}$ > 54−60 ml min kg^−1^ and were involved in endurance-based activities at least 3 times/week), respectively. In each panel, the grey shape highlights the slope, running speed or exercise duration used in the majority of studies (ranging between the 25th and 75th percentile). In the cases where data were not fully presented in the manuscript, data were extracted from original figures using ImageJ software (ImageJ V.1.45 s, National Institute of Health, MD, USA)

When analyzing EIMD markers (Fig. 2), it is also important to consider the methodological differences between DR protocols. Slope, duration, running speed as well as the experimental design (i.e. continuous DR *vs* intermittent DR) may influence the magnitude of EIMD. Overall, in previous studies, for trained and untrained populations, the median (*Q*_1_; *Q*_3_) slope, exercise duration, and running speed were − 12.0% (− 15.0%; − 10.0%), 40 min (30 min; 45 min) and 11.3 km h^−1^ (9.8 km h^−1^; 12.9 km h^−1^), respectively. More specifically, in studies assessing isometric MVC in response to DR, the median (*Q*_1_; *Q*_3_) slope was − 14.95% (− 15.0%; − 12.0%, Fig. 2a). In studies assessing CK, and DOMS following DR, the median (*Q*_1_; *Q*_3_) slope was − 10% (− 15.0%; − 10.0%) and − 12.8% (− 15.8%; − 10.0%), respectively (Fig. 2b, c). The median (*Q*_1_; *Q*_3_) running speed was 11.5 km h^−1^ (10.1 km h^−1^; 12.9 km h^−1^), 11.3 km h^−1^ (9.7 km h^−1^; 12.5 km.h^−1^), 12.3 km.h^−1^ (9.7 km.h^−1^; 14.6 km.h^−1^) in studies analysing isometric MVC, CK and DOMS, respectively (Fig. 2d–f). Finally, the median (*Q*_1_; *Q*_3_) exercise duration in DR protocols was 40 min (30 min; 40 min), 30 min (30 min; 45 min), 40 min (30 min; 46.25 min), in studies analysing isometric MVC, CK and DOMS, respectively (Fig. 2g–i). Moreover, there was an important proportion of intermittent protocols in untrained compared to trained populations (48.0% vs. 21.1% on average, respectively), likely to facilitate the completion of DR exercises in people not accustomed to eccentric muscle actions. The results also highlight important discrepancies between studies in the assessment of DOMS (i.e. methods of evaluation, muscles and/or muscle groups investigated, type of scale used) limiting the amount of data presented in Figs. 1c and 2c, f, i. We can also notice a large variability in blood CK elevation in response to DR among studies in untrained and trained populations. Sex, age, genotype, menstrual cycle, familiarisation with DR and DR characteristics could explain this variability in blood CK response among studies. While blood CK elevation is considered a reliable and indirect marker of EIMD, comparing the extent of EIMD between studies using this marker seems impossible due its variable nature between conditions and individuals.

**Fig. 3:**
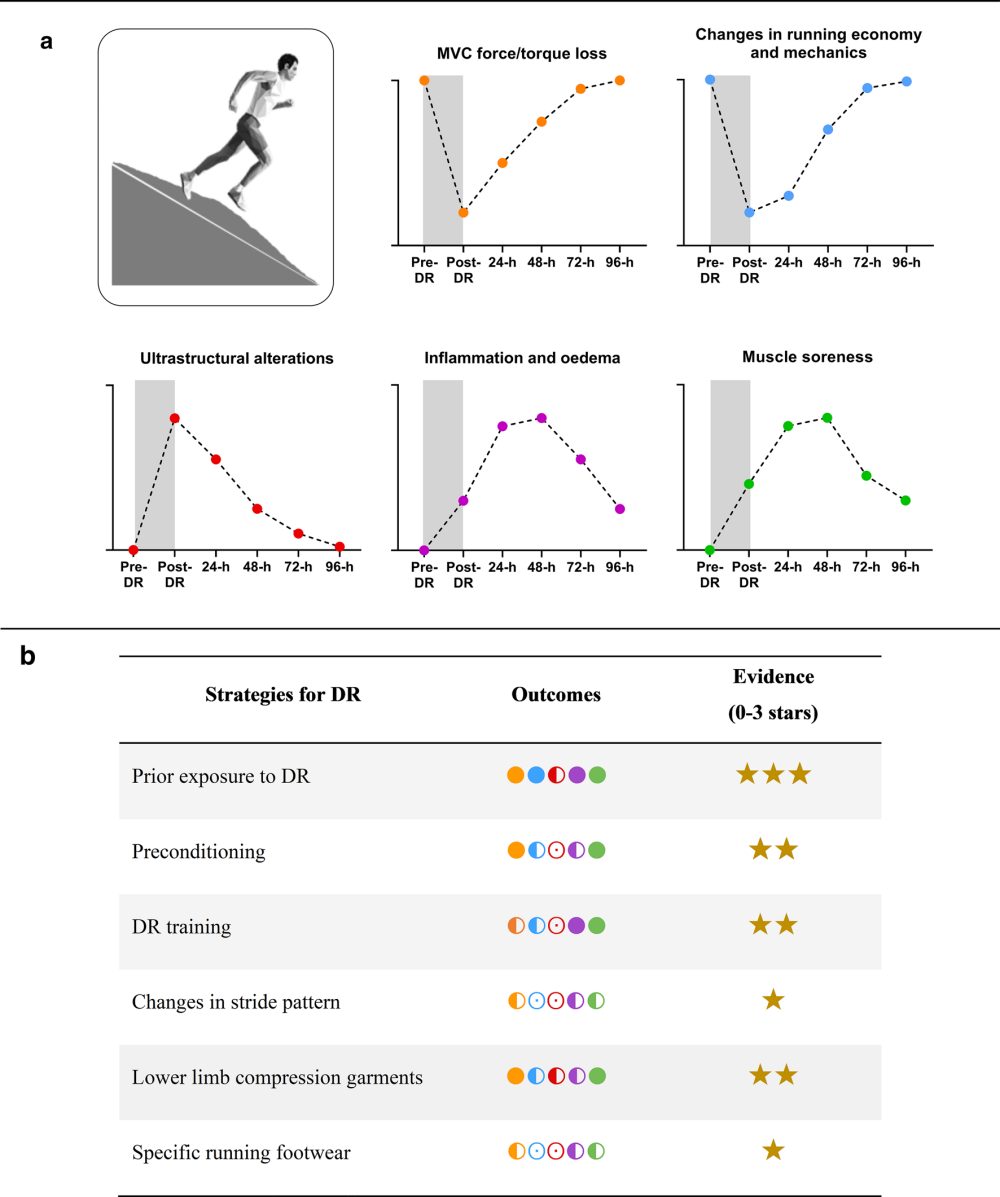
**a** Schematic representation of the time course of alterations following downhill running (DR) and **b** current scientific evidence on the benefits of different adaptation strategies to DR (i.e. prior exposure to DR [46, 63, 67, 68, 85, 92, 121, 141, 143, 165, 181, 184, 185, 192, 193, 196, 197], preconditioning strategies [16, 81], DR training [14, 209, 210, 254], changes in stride pattern [32, 67, 68, 218, 219, 221, 220, 228, 255], the use of lower limb compression garments [15, 236, 237], and the use of specific footwear [28, 149, 253, 256]. In **a** and **b**, orange, blue, red, purple, and green spheres correspond to isometric MVC force/torque loss, changes in running economy and mechanics, ultrastructural alterations, inflammation and oedema, and muscle soreness, respectively. In **b**, full sphere, indicates a high tendency for a beneficial effect; half full sphere, indicates a lack of tendency for a beneficial effect and/or lack of studies for this parameter; empty sphere, indicates no data for this parameter. In b for each strategy, the strength of scientific evidence is represented with stars on a 1–3 scale where 1 meaning little evidence and 3 meaning high evidence. The level of evidence was determined according to the abundance of data available and the number of studies reporting the same outcome. The illustration of the running man is adapted from ©maximmmmum/Adobe Stock

Manipulating DR characteristics, independently or not, may lead to varied extents of EIMD. For example, increasing the slope by 7.0% in combination with a higher speed (+ 4.5 km h^−1^) induced a greater reduction in MVC torque and blood CK elevation following a 45 min DR [38]. Nevertheless, knowing the DR characteristics that have been used the most in scientific studies so far may help orientate future protocols using DR models. Specifically, from the analysis presented in Fig. 2, where isometric MVC, blood CK and DOMS were investigated in response to DR, it becomes clear that high running speeds (> 15 km h^−1^), steeper slopes (> 15%) and longer exercise durations (> 60 min) remain to be investigated. Future studies should also aim to isolate the different characteristics of DR to understand whether one or a combination of them might induce more EIMD than others.

### Other Factors Potentially Influencing Muscle Damage Following Downhill Running

Few studies have investigated the influence of sex on indirect markers of muscle damage following DR [10, 67, 147, 168, 191]. Following a 30 min DR (slope: − 20%; speed: 10.0 km h^−1^), no differences were observed in MVC torque loss and no alterations in the central component of neuromuscular fatigue were observed between females and males [10]. Although the magnitude of absolute (compared to baseline value) blood CK elevation is generally similar between males and females following DR [147, 168], Oosthuyse et al. [168] reported a faster return to baseline in females compared to males. It was suggested that an elevated oestrogen level could confer a protective effect against EIMD [148] and/or may reduce the secondary phase of EIMD caused by local inflammation [161]. Carter et al. [141] also reported lower plasma CK elevation in link with higher oestrogen contents after 30-min DR (slope: − 10%; intensity: 60% $\dot{V}{\text{O}}_{{{\text{2max}}}}$). In contrast, although blood CK and DOMS elevations generally occur concomitantly in males, this does not seem to be the case in females. The DOMS response following DR was delayed in females and may be influenced by the menstrual phase (with a greater recovery rate during the mid-luteal phase) [168], though this remains to be confirmed with additional studies.

Many studies have investigated adaptations to DR in adult populations, but only a limited number have included adolescents, pre-pubescent children and older populations. Some studies did not report differences in indirect markers of muscle damage between pre-pubescent children and adults [191], or between young and older trained triathletes [180] after DR. Other studies reported trends towards higher levels of indirect markers of muscle damage (neutrophil counts, plasma CK and DOMS) in young men compared to older men after DR [8, 142] but with comparable recovery kinetics [8]. Additional studies are required to better understand the effect of maturation and/or age on the physiological responses to DR.

## Adaptation Strategies to Downhill Running

As discussed in Sects. 2 and 3 of this review, the occurrence of EIMD following DR may be deleterious to running performance and subsequent recovery. The search for adaptation strategies to DR, i.e. strategies to better tolerate repeated eccentric muscle actions, is, therefore, warranted. Applications extend from recreational to competitive sport involving DR, such as trail and road running. To date, the focus has been on either pre-exercise strategies designed to accustom the athlete to the demanding nature of DR, or in-situ strategies to reduce the occurrence of EIMD (Table 1 and Fig. 3). Pre-exercise strategies (i.e. preconditioning, DR training) involve a first exposure to eccentric muscle actions prior to DR. In-situ strategies mainly consist in wearing innovative running apparels (i.e. compression garments, specific footwear) or voluntarily changing one’s running pattern during DR.

**Table 1:** Summary of studies examining adaptation strategies to downhill running (DR), i.e. prior exposure to DR, preconditioning strategies, DR training, changes in stride pattern, the use of lower-limb compression garments and the use of specific footwear

Study	Participants	Downhill run			Interventions	Main outcomes
		Duration	Intensity	Slope (%)		
Prior exposure to DR						
Byrnes et al. [193]	College students (*n* = 22; 11 males and 11 females)	30 min	Speed corresponding to 170 bpm recorded on level grade	− 17.6	Bout 2 post 3, 6 or 9 weeks	+ DOMS post 3 and 6 weeks + Blood CK and Mb post 3 and 6 weeks ↔ DOMS and plasma CK post 9 weeks
Pierrynowski et al. 192]	Healthy male students (*n* = 9)	3 × 4 min, *r*’=5 min	60% $\dot{V}$O_2max_	− 10	Bout 2 post 4 days	+ DOMS ↔ MVC torque of KE and knee flexors (concentric muscle actions)
Westerlind et al. 121]	Recreational active adults (*n* = 7; 4 males and 3 females)	30 min	40% $\dot{V}$O_2peak_	− 10	Bout 2 post 2 weeks	+ DOMS + Blood CK elevation + $\dot{V}$O_2_ drift during DR
Westerlind et al. [141]	Recreational active adults (*n* = 6; 3 males and 3 females)	45 min	50% $\dot{V}$O_2max_ (9.7 km h^−1^ on average)	− 10	Bout 2 post 2 weeks	+ DOMS + Blood CK elevation ↔ $\dot{V}$O_2_ drift during DR ↔ Muscle and rectal temperature ↔ Stride length and frequency
Smith et al. [143]	Active male adults (*n* = 9)	3 × 15 min, *r*’ = 5 min	70% s$\dot{V}$O_2peak_ recorded on level grade	− 16.2	Bout 2 post 2 weeks	+ DOMS + Blood CK elevation ↔ Neutrophil count during the post 12-h
Eston et al. [67]	Active adults (*n* = 18; 9 males and 9 females)	5 × 8 min, *r*’ = 2 min	11.3 km h^−1^	− 12	Bout 2 post 5-weeks at − 8% PSF, + 8% PSF or PSF	+ Blood CK elevation + Muscle tenderness ↔ MVC torque decrement of KE ↔ Gender effect
Rowlands et al. [68]	Active male students (*n* = 18)	9 × 5 min, *r*’ = 2min	10.5 km h^−1^	− 15	Bout 1 at − 8% PSF, PSF or + 8% PSF Bout 2 post 2 weeks at PSF	+ DOMS + MVC force decrements of KE (PSF and + 8% PSF) ↔ MVC force decrement of KE (− 8% PSF)
McKune et al. [46]	Healthy male adults (*n* = 11)	60 min	75% s$\dot{V}$O_2peak_ estimated on level grade	− 13.5	Bout 2 post 2 weeks	+ Blood CK elevation + Inflammatory response (e.g. higher clearance)
Chen et al. [92]	Healthy male students (*n* = 12)	30 min	70% $\dot{V}$O_2max_	− 15	Bout 2 post 5 days	+ DOMS + Blood CK elevation + RE changes (expressed as $\dot{V}$O_2_) with faster recovery time ↔ MVC torque decrement
Smith et al. [143]	Active male adults (*n* = 6)	60 min	75% s$\dot{V}$O_2max_ estimated on level grade	− 13.5	Bout 2 post 2 weeks	+ Blood CK elevation + DOMS + Systemic inflammation
Smith et al. [184]	Healthy male adults (*n* = 11)	60 min	75% s$\dot{V}$O_2max_ estimated on level grade	− 13.5	Bout 2 post 2 weeks	+ Blood CK elevation + DOMS ↔ Neutrophil count during the post 24-h
Green et al. [63]	Recreational adult female athletes (*n* = 10)	6 × 5 min, *r*’ = 2 min	13 km h^−1^	− 12	Bout 2 post 2 weeks	↔ Faster recovery of MVC torque of KE ↔ Blood CK elevation ↔ DOMS ↔ Insulin and glucose response
Park et al. [181]	Moderately trained adults (*n* = 12; 9 males and 3 females)	40 min	70% $\dot{V}$O_2max_ (14.9 km h^−1^ on average)	− 10	Bout 2 post 3 weeks	+ Blood CK elevation + Apoptic response
Park and Lee [165]	Moderately trained male adults (*n* = 13)	40 min	70% $\dot{V}$O_2max_	– 10	Bout 2 post 3 weeks	+ Blood CK elevation + Oxidative stress
He et al. [85]	Moderately trained males (*n *= 22), Placebo group	40 min	65–70% $\dot{V}$O_2max_	– 10	Bout 2 post 2 weeks	+ DOMS + Blood CK elevation
Dolci et al. [197]	Physically active males (*n* = 9)	60 min	65% $\dot{V}$O_2max_	− 10	Bout 1 and 2 followed by 40-min HS level running. Bout 2 post 2 weeks	+ MVC torque decrement of KE + DOMS + Physiological strain (e.g. rectal temperature) during subsequent HS exercise + Perceived exertion and thermal sensation during subsequent HS exercise
Tuttle et al. [196]	Physically active males (*n *= 6)	30 min	Lactate threshold (66% $\dot{V}$O_2max_ on average)	− 10	Bout 1 and 2 in HS condition Bout 2 post 1 week	+ DOMS + Physiological strain (e.g. rectal temperature) + Cellular stress response and thermotolerance
Preconditioning strategies						
Eston et al. [16]	Healthy male students (*n* = 10)	5 × 8 min, *r*’ = 2 min	80% HR_max_	− 10	100 KE maximal eccentric contractions 2 weeks prior DR	+ Blood CK elevation + DOMS + MVC torque of KE (concentric and eccentric muscle actions) ↔ Muscle tenderness
Lima et al. [81]	Young males (*n* = 30)	30 min	70% $\dot{V}$O_2max_ (10.2 km h^−1^ on average)	− 15	10 KE isometric MVCs 2-day prior DR	+ Faster recovery of KE MVC torque + DOMS + Blood CK elevation ↔ $\dot{V}$O_2_ drift during DR and post RE changes (expressed as $\dot{V}$O_2_)
Downhill running training						
Schwane et al. [14]	Healthy adults (*n* = 50; *n* = 20 in DR groups; 7 males and 13 females)	9 × 5 min, *r*’ = 2 min	80% s$\dot{V}$O_2max_ recorded on level grade (9.4 km h^−1^ on average)	− 10	5 sessions/week for 1 or 2 weeks	+ DOMS (especially with 2-weeks training) + Blood CK elevation
Law et al. [254]	Military young males (*n* = 18)	30 min	50-60% $\dot{V}$O_2peak_ (12.9km h^−1^ on average)	− 10	1 session/week for 8 weeks	+ DOMS + Blood CK elevation
Shaw et al. [210]	Highly trained male adults (*n* = 19)	15–45 min (divided into 3 different intensity stages)	90, 100 and 110% speed at lactate turn point	− 5	2 sessions/week for 8 weeks in addition to their habitual training program	↔ Velocity at lactate turn point ↔ RE improvement (expressed as energy cost)
Toyomura et al. [209]	Healthy male adults (*n* = 18)	5–20 min	At lactate threshold (14.9 km h^−1^ on average)	− 10	3 sessions/week for 5 weeks	+ MVC torque of KE (isometric, concentric and eccentric muscle actions) + Change of direction ability + Little effect on the aerobic capacity
Change in stride pattern						
Eston et al. [67]	Active adults (*n* = 18; 9 males and 9 females)	5 × 8 min, *r*’ = 2 min	11.3 km h^−1^	− 12	Bout 1 at PSF, Bout 2 post 5 weeks, at − 8% PSF, PSF or + 8% PSF	↔ Muscle tenderness ↔ Blood CK elevation ↔ MVC force decrement of KE
Rowlands et al. [68]	Active male students (*n* = 18)	9 × 5 min, *r*’ = 2 min	10.5 km h^−1^	− 15	Bout 1 at − 8% PSF, PSF or + 8% PSF, Bout 2 post 2 weeks, at PSF	− DOMS (overstride–Bout 1) + MVC force/torque of KE (understride–Bout 1) + Greater RBE with overstride (especially for DOMS)
Snyder and Farley [32]	Male adult runners (*n* = 9)	5 min, *r*’ = 5 min	7.3 km h^−1^	− 5.2	At PSF, 85% PSF, 92% PSF, 108% PSF and 115% PSF	↔ RE (expressed as net metabolic cost)
Sheehan and Gottschall [220]	Healthy young adults (*n *= 10; 5 males and 5 females)	2 × 5 min	10.8 km h^−1^	− 10.5	At PSF, + 15% PSF and − 15% PSF	− RE at + 15% PSF and − 15% PSF (expressed as $\dot{V}$O_2_)
Giandolini et al. [255]	Male adults trained in trail running (*n* = 23)	6.5 km	As fast as possible (13.3 km h^−1^ on average over analysed sections)	− 16.8 on average	Anterior vs. posterior FSP	FSP differently affects the components of impact shock acceleration: ↔ Adopting a more anterior FSP leads to improve attenuation of axial and resultant impact-related frequencies
Giandolini et al. [219]	Male adults trained in trail running (*n* = 23)	6.5 km	As fast as possible (13.3km h-^−1^ on average over analysed sections)	− 16.8 on average	Anterior vs. posterior FSP	Anterior FSP: − MVC torque decrement in KE − Peripheral component of neuromuscular fatigue in KE High FSP variability: + MVC torque decrement of KE and PF + Peripheral component of neuromuscular fatigue of KE
Vincent et al. [221]	Experienced adult runners (*n* = 40; 23 males and 17 females)	40-min (including warm-up, 6 trials and a cool-down)	10.6 km h^−1^ on average	− 10.5	DR trials at PSF, + 10% PSF, + 5% PSF, − 5% PSF and –− 10% PSF	+ ± 5% PSF: energy conservation and protection of lower extremity joints − Extended periods at − 10% PSF: may increase relative intensity of running, loading impulses and force attenuation by lower extremities
Vernillo et al. [228]	Male adults experienced in trail running (*n* = 14)	2.5-h graded run (with 3 × 20min DR)	As fast as possible over the graded run (9.5 km h^−1^ on average)	− 15	Manipulation of FSP (every 30-s during DR)	↔ Extent of biomechanical changes ↔ Neuromuscular fatigue component ↔ Energy cost drift
Baggaley et al. [218]	Active adults (*n* = 19; 9 males and 10 females)	< 15 s	11.9 km h^−1^	− 8.8 and − 17.6	At PSF, + 10% PSF, and − 10% PSF	PSF manipulation: + Lower extremity energy absorption + Impact attenuation + Shorter PSF: reduce the demand placed on the knee and hip, and may aid in reducing EIMD and improving performance − Longer PSF: greater energy absorption
Use of lower-limb compression garments						
Webb and Willems [236]	Healthy male adults (*n* = 18)	5 × 8 min, *r*’ = 2 min	80% $\dot{V}$O_2max_	− 10	Average compression rate of 18mmHg at the calf and 9mm Hg at the thigh	+ DOMS at post-24 h and 48 h + Jump height recovery
Valle et al. [237]	Male adult amateur soccer players (*n* = 15)	40 min	73% $\dot{V}$O_2max_ (10.7 km h^−1^ on average) $\dot{V}$O_2max_ measured with a + 3% slope	− 10	Smallest size of garment participants could wear	+ − 26.7% EIMD (presence of albumin in muscle fibres)
Ehrström et al. [15]	Male adults well-trained in trail running (*n* = 13)	40 min	55% $\dot{V}$O_2max_ (15.1 km h^−1^ on average) $\dot{V}$O_2max_ measured with a + 10% slope	− 15	Average compression rate of 18 mmHg to the calf and 21 mm Hg at the thigh	+ Attenuation of KE soft-tissue vibration + Neuromuscular fatigue component and recovery + MVC torque decrements in KE and recovery + DOMS ↔ RE (expressed as $\dot{V}$O_2_)
Use of specific running footwear						
Hardin and Hamill [149]	Adult male recreational runners (*n* = 24)	30 min	12.2 km h^−1^	− 12	Soft vs medium vs hard midsole shoes	↔ Leg shock ↔ Haematological responses
Lussiana et al. [28]	Adult male recreational runners (*n* = 14)	7 × 5 min (DR, level and uphill), *r*’ = 5 min	10 km h^−1^	–8, −5 and − 2 for DR	Minimal shoes vs traditional shoes	+ RE (expressed as cost of running) when all slopes are considered ↔ RE (expressed as cost of running) for negative slopes only
Lussiana et al. [256]	Adult male recreational runners (*n* = 14)	7 × 5 min, *r*’ = 5 min	10 km h^−1^	− 8	Minimal shoes vs traditional shoes	+ With minimalist shoes: leg compression, contact time, vertical displacement of the centre of mass (vertical and horizontal leg stiffness respond differently to change in footwear)
Chan et al. [253]	Adult regular distance runners (*n* = 27; 15 males and 12 females)	5 min	8.3 km h^−1^ on average	− 10	Maximalist shoes vs traditional running shoes	− With maximalist shoes might not reduce the external impact loading. Instead, it may increase the external impact loading during DR

DOMS, delayed onset muscle soreness; EIMD, exercise-induced muscle damage; FSP, foot stride pattern; HS, heat stress (30° C); KE, knee extensors; MVC, maximal voluntary contraction (isometric muscle actions unless otherwise stated); PSF, preferred stride frequency; RBE, repeat bout effect; RE, running economy; *r*’, recovery duration; $\dot{V}{\text{O}}_{{{\text{2max}}}}$, maximal aerobic capacity measured on level grade unless otherwise stated; s$\dot{V}{\text{O}}_{{{\text{2max}}}}$, running speed associated with $\dot{V}{\text{O}}_{{{\text{2max}}}}$; + , positive effect; −, negative effect; ↔ , no effect

### Pre-exercise Adaptation Strategies

#### Prior Exposure to Downhill Running

Performing two bouts of DR separated by several days or weeks is well-known to reduce or attenuate markers of muscle damage after the second bout [192, 193]. This protective mechanism is termed as ‘repeated bout effect’ (RBE) [57, 194, 195]. Several mechanisms were identified to be likely involved in the RBE, including neural adaptations (e.g. ɑ-motoneuron excitability, shift in motor unit recruitment), adaptations of the muscle–tendon complex (e.g. reduced fascicle elongation, increased tendon compliance), increased sensitivity to inflammation and improved muscle extracellular matrix remodelling [190]. Byrnes et al. [193] reported smaller increases in plasma CK, plasma myoglobin and DOMS up to 48 h after a second DR bout when two 30 min bouts (slope: − 10%; intensity: 170 beats min^−1^ of heart rate recorded on level grade) were separated by 3-, 6- and 9-weeks. Further studies corroborated these results by reporting a lower leakage and/or a faster clearance of intracellular muscle proteins in the blood [46, 63, 67, 85, 92, 121, 141, 143, 165, 181, 185], lower DOMS [63, 68, 85, 92, 121, 141, 143, 185, 192, 196, 197] and enhanced recovery of MVC force/torque [63] after a second DR bout (Table 1 and Fig. 3b). The RBE is generally reported in greater proportion through blood CK elevation than DOMS and post-exercise MVC force/torque decrement [190]. Chen et al. [92] also suggested a beneficial effect of a first DR bout on RE (expressed as $\dot{V}{\text{O}}_{{2}}$; preserved up to 72 h post exercise) and stride parameters (preserved stride length and frequency) recorded after the second bout, likely explained by less muscle damage after the second bout. It can be hypothesized that a lower magnitude of EIMD following a second bout of DR may limit the increase in neural input to the muscle (especially during the push-off phase of the running cycle) resulting in a lower increase in $\dot{V}{\text{O}}_{{2}}$_,_ and consequently lower alterations in RE. It would be interesting to analyse simultaneously the electromyographic activity of KE and PF and RE during the completion of two bouts of DR separated by several days or weeks.

Prior exposure to DR confers protective effects against EIMD that are limited in time. Byrnes et al. [193] showed that the RBE procured by a prior 30 min DR disappeared after 9 weeks with no eccentric muscle actions between the first and second bout. The magnitude of the RBE would mainly depend on the level of exposition to EIMD, with the most EIMD leading to the highest RBE [190, 198]. Within this context, implementing specific DR sessions in the training program of runners could be recommended to benefit from the protective effects of the RBE and adapt well to the next DR session or to better tolerate DR sections in off-road or road races.

#### Preconditioning Strategies

Performing an isolated strenuous exercise within days prior to a DR session, termed as ‘preconditioning exercise’ may also confer a form of RBE [16, 81]. Eston et al. [16] investigated the effects of repeated isolated KE eccentric muscle actions (100 continuous voluntary eccentric muscle actions at low speed: 0.58 rad s^−1^) performed 14 days prior to a 40 min DR bout (5 × 8 min interspersed with 2-min rest; slope: − 10%;intensity: 80% maximal heart rate). Compared to the control group, the reduction in maximal isokinetic peak torque (concentric and eccentric at 2.82 rad s^−1^ and 0.58 rad s^−1^) was less pronounced at lower eccentric and concentric muscle action speeds (− 22 and − 19% immediately post-DR, respectively), accompanied by a lower elevation in plasma CK (− 79% 48-h post-DR) and an increase in muscle tenderness (− 52% 48 h post-DR) up to 7 days post-DR.

A short isometric preconditioning exercise (10 MVCs at long muscle-length performed 2 days prior DR) could also confer RBE [81]. The authors reported beneficial effects of isometric preconditioning on MVC torque recovery (return to baseline 72-h post in the preconditioning group *vs* no full recovery 96-h post in the control group) and DOMS (− 25% 48-h post in the preconditioning group *vs* control group). However, no beneficial effects of isometric preconditioning were observed on RE (expressed as $\dot{V}{\text{O}}_{{2}}$), counter movement jump height and plasma CK [81]. Isometric preconditioning exercise at long muscle length could confer RBE, but in lower proportion compared to eccentric preconditioning, although these data are based on studies investigating adaptations of upper-limb muscles [199, 200]. Implementing isometric instead of eccentric-based exercises in training programmes could be an interesting strategy for runners aiming to benefit from the RBE but with no prior muscle damage, especially in the lead up to competitions.

#### Downhill Running Training

Eccentric-biased exercise training is well known to enhance maximal strength production [201, 202]. In endurance runners, resistance training has been proposed as a strategy to improve running economy [203, 204]. Concurrent plyometric and endurance training for several weeks (8–12 weeks) improved RE (expressed as $\dot{V}{\text{O}}_{{2}}$; + 4 to + 8% recorded on level grade) [205–208]. Thus, due to their eccentric-biased muscle action modality, repeated DR sessions may also stimulate muscle growth factors, promote adaptations to the neural system, muscle–tendon complex and running biomechanics, and could lead to improvements in RE. Toyomura et al. [209] argued in favour of DR training to improve running performance on level grade. The authors suggested that DR training could improve the effectiveness of the stretch–shortening cycle (paramount for level running performance; [128]) by accentuating the eccentric work (impact and stance phase) involved, which may consequently enhance RE on flat sections. Further studies are required to verify if DR training may lead to a better RE on negative slopes.

In addition, DOMS recorded after a 45-min DR bout was lower following a short DR training (five training sessions in 1 week; duration: 5–15 min; slope: − 10%; intensity: 80% of running speed associated with $\dot{V}{\text{O}}_{{{\text{2max}}}}$ recorded on level grade, s$\dot{V}{\text{O}}_{{{\text{2max}}}}$, with % s$\dot{V}{\text{O}}_{{{\text{2max}}}}$ corresponding to running speed associated to a percentage of $\dot{V}{\text{O}}_{{{\text{2max}}}}$) compared to a non-trained control group [14]. In the same study, a longer training period (ten training sessions in 2 weeks vs. five training sessions in 1 week) conferred a greater beneficial effect on DOMS after a 45-min DR compared to the control group. An improvement in maximal isometric and isokinetic (concentric and eccentric) torque (+ 9 to + 24%) was also recorded after a 5-week DR training in physically active young males (three sessions per week; duration: 5–20 min; slope: − 10%) [209]. In contrast, the same authors did not report any effect of DR training on $\dot{V}{\text{O}}_{{{\text{2max}}}}$, running speed at $\dot{V}{\text{O}}_{{{\text{2max}}}}$ and RE (expressed as energy cost) in physically young men. This result was supported by Shaw et al. [210], who reported no change in RE (expressed as energy cost) and stride parameters in already well-trained runners taking part in an 8-week DR training program (with two DR sessions per week added to their habitual training; duration: 15–45 min; slope: − 5%; speed: from 90 to 110% speed at lactate threshold). It can be hypothesized that the initial training level may lower the effectiveness of DR training on RE responses during level running and/or DR. In this regard, Breiner et al. [211] showed that the most economical runners in level running were also the most economical in uphill and downhill slopes, reinforcing the importance of running experience in the RE responses to DR training. It is well-established that at least a single bout of DR can confer a protective mechanism against EIMD. Although the literature is scarce on the load-response to DR training, it seems that a greater training volume could confer a greater protective effect against EIMD [14]. Accordingly, DR training could be incorporated in the training regime of off-road and road runners, in an attempt to improve tolerance to DR, and possibly enhance running performance in races. Recent data tend to support these recommendations showing that maximal strength and lower-limb musculotendinous stiffness as well s$\dot{V}{\text{O}}_{{{\text{2max}}}}$ are strong predictors of DR performance in trail runners used to DR [212]. These results strengthen the rationale for including specific DR sessions in the athlete’s training regime. In addition, DR training may improve the effectiveness of the stretch–shortening cycle on level grade [128]. On another hand, caution must be taken, and a principle of progressivity must be applied when implementing DR sessions into a runner’s training program to avoid maladaptation and injuries. The greater negative net joint work accompanied by larger extension moments and negative joint power [34] reported in DR might require longer recovery times post training sessions, especially at the beginning of the implementation. Further studies should aim to investigate the load-response to DR training to provide practical guidance for runners. From a scientific standpoint, training data suggest that implementing more than one familiarisation session to DR is crucial in experimental cross-over studies (e.g. [15, 213]) using the DR model to investigate *in-situ* and recovery strategies, so that the RBE can be offset.

### In-situ Adaptation Strategies

#### Optimisation of Stride and Foot Strike Pattern

The manipulation of foot stride (i.e. length and frequency) and foot strike pattern (i.e. rear-, mid- and fore-foot) can influence the mechanical strain applied to lower limb muscles during running [214–217]. Accordingly, manipulating foot stride frequency [218] and foot strike pattern [219] during DR could reduce EIMD including neuromuscular fatigue. Following an intermittent 45-min treadmill DR (9 × 5-min; slope: − 15%; speed: 10.5 km h^−1^), Rowlands et al. [68] reported lower MVC torque decrements post-DR using an understride strategy (− 8.8% MVC torque; 92% preferred stride length) compared with an overstride (− 14.7% MVC torque; 108% preferred stride length) or preferred stride strategy (− 15.5% MVC torque). This was associated with a faster recovery of muscle function within days following exercise. Interestingly, the authors also reported a greater RBE conferred by a first bout of DR with the overstride strategy, likely caused by more EIMD (+ 20 to + 40 mm for DOMS measured on a visual analogue scale immediately post- and 24-h post exercise, respectively) compared to understride and preferred stride strategies. In contrast, Eston et al. [67] observed no difference in plasma CK, MVC force and muscle tenderness after a 40-min intermittent DR (5 × 8-min; slope: − 12%; speed: 11.3 km h^−1^) in untrained participants, regardless of the foot stride manipulation tested. Regarding the impact of foot stride manipulation on running performance, Sheehan et al. [220] reported that foot stride manipulation can affect RE (expressed as energy cost) during DR (slope: − 10.5%; speed: 10.8 km h^−1^) with a lower energy cost reported with a preferred stride frequency (PSF) compared to understride (− 12% for 85% PSF) and overstride (− 6% for 115% PSF). Similar results, albeit not significant, were reported by Snyder and Farley [32] when PSF was compared to understride (− 17.4% vs. 85% PSF) and overstride (− 16.2% vs. 115% PSF) during DR at shallow negative slope and low running speed (− 5.2%; speed: 7.2 km h^−1^). Recently, Vincent et al. [221] corroborated previous results by showing that slight changes in PSF (± 5%) during DR had no detrimental effect on the energy cost of running. In addition, the choice of stride length affects the energy absorption and impact attenuation characteristics of lower extremity joints during DR [218]. More precisely, the authors reported that shortening step length by 10% (i.e. using an overstride strategy) reduced knee joint (at a slope corresponding to − 8.8% and − 17.6%) and hip energy absorption (at a slope corresponding to − 8.8%) and enhanced impact attenuation. Such a strategy may be applied to reduce EIMD and maintain running performance [218]. It is worth mentioning that, on level grade, novice runners ran with a PSF that was about 8% lower than their optimal stride frequency (established from polynomial equations with stride frequency, $\dot{V}{\text{O}}_{{2}}$ and HR) whereas the PSF of experienced and trained runners was significantly closer to their optimal one (~ − 3%) [222]. Taken together, these data suggest that during DR: (1) metabolic cost, energy absorption and impact attenuation depend on the stride pattern; (2) adopting a stride pattern close to PSF may be an effective strategy to optimize RE. On another hand, the benefits of increasing stride frequency on energy absorption and impact attenuation may occur at the expense of RE. Additional studies are necessary to clarify the effects of stride manipulations on RE and subsequent EIMD during DR, while also taking into account different training levels.

By influencing muscle-length and muscle activity during the ground-contact phase, foot strike pattern could also influence the occurrence of EIMD. Depending on the portion of the foot that initially hits the ground [223], three main foot strike patterns employed by runners have been identified: rearfoot (heel contacts first), midfoot (metatarsal heads contact first accompanied by heel contact) and forefoot (metatarsal heads contact first without heel contact). The forefoot pattern is characterized by greater plantar and knee flexion at initial contact [224–226]. The negative work (i.e. the work done to decelerate the body’s centre of mass with respect to the environment) has been shown to be lower at the knee and greater at the ankle in forefoot compared to rearfoot strike [227]. In line with these observations, Giandolini et al. [219] investigated the effects of foot strike pattern on muscle activity and muscle fatigue following a 6.5 km off-road DR (average slope: − 16.8%) in well-trained trail runners. In this study, forefoot strike was associated with higher activity of the *gastrocnemius lateralis* and lower activity of the *vastus lateralis* and *tibialis anterior* during DR. It was also positively correlated with higher decrements in the peripheral fatigue components (i.e. twitch and 10 to 100 Hz ratio decrements) compared to rearfoot strike [219], which may potentially accentuate damage in PF muscles. Interestingly, runners exhibiting various foot strike patterns (alternation between forefoot, midfoot and rearfoot) during DR presented lesser peripheral alterations underlying neuromuscular fatigue following the run. Although no ideal foot strike pattern seems to exist, Giandolini et al. [219] suggested that a high variability in foot strike pattern (based on regression analysis) may limit neuromuscular fatigue during DR. However, when this strategy (i.e. voluntarily alternating foot strike patterns every 30 s) was applied in DR sections over a 2.5-h graded treadmill run, no benefit was reported in terms of neuromuscular alterations compared to the control group (i.e. no switch pattern over DR sections), though no marker of EIMD was recorded in this study [228]. It is possible that (1) the beneficial effect of the variability in foot strike pattern during DR sections was offset by the extent of neuromuscular fatigue during the uphill and level sections (both performed with natural foot strike for 60 min and 20 min in total, respectively), (2) a longer training/familiarisation period is required to benefit the most from this strategy. In the field, we can assume that the best runners spontaneously adapt their running pattern, technique and propulsive function to the varied characteristics of the terrain, which would influence the neuromuscular, energetic and biomechanical demands, thus limiting direct comparisons with laboratory studies.

#### The Use of Lower Limb Compression Garments

Although wearing lower limb compression garments (CGs) after exercise has been shown to facilitate the recovery process [229–231], there is currently no consensus regarding their use during exercise [15, 232–235]. To date, only a few studies have investigated the impact of wearing CGs during DR [15, 236, 237]. Ehrström et al. [15] showed that wearing high-pressure compression garments (15–20 mmHg) during 40-min treadmill DR (slope: − 15%; intensity: 55% $\dot{V}{\text{O}}_{{{\text{2max}}}}$) likely presented benefits for reducing alterations in the peripheral and central components of neuromuscular fatigue (for KE muscles) immediately after DR, in well-trained trail runners. The beneficial effects of CGs may be explained by an attenuation of soft-tissue vibrations which might improve muscle function [238], accompanied by lower muscle activity during exercise [15, 239]. Ehrström et al. [15] suggested that wearing CGs may exert ‘dynamic immobilization’, reducing soft-tissue oscillation and improving joint stability, and in turn, enhancing neural input [240, 241]. Through a histological and immunohistochemical analysis of muscle biopsy samples of KE muscles, Valle et al. [237] reported less muscle fibre injuries (− 26.7% on average) after 40-min DR (slope: − 10%) for the compressed leg compared to the control leg in amateur soccer players. However, since soccer players were not accustomed to DR, this raises the question of the extent of the protective effect conferred by CGs in trained athletes used to running on DR sections. In addition, CGs might contribute to enhanced post-exercise muscle recovery [15, 239]. A greater mechanical protective effect was also observed during the recovery period in KE compared to PF muscles only when athletes wore CGs during DR. This suggests a beneficial mechanical support particularly for the largest muscles recruited in DR [15]. In summary, these few data suggest that wearing CGs could be a relevant strategy to limit EIMD during specific off-road and/or road races involving notable DR sections. Recreational runners and masters athletes (> 40 years) more prone to EIMD may also benefit the most from CGs to help them to stabilise their muscle mass during DR. Wearing CGs could also facilitate adherence to training by limiting the risk of muscle–tendon unit injuries thanks to a reduction in impact shock accelerations during long duration running sessions [15, 242].

#### The Use of Specific Running Footwear

The selection of appropriate running footwear has become an essential requirement for distance running [243]. Recent meta-analyses and systematic reviews showed that footwear characteristics (i.e. shoe mass, cushioning, motion control, midsole viscoelasticity, comfort, longitudinal bending stiffness, drop height) may reduce the risk of injury [244–246] and improve RE [247, 248] on level grade. Minimalist shoes (i.e. lighter mass, greater sole flexibility, lower drop height, smaller heel elevation) have been shown to induce beneficial biomechanical adjustments (e.g. accentuated mid-forefoot stride pattern, knee and ankle range of motion changes) during level running compared to traditional shoes [246, 249]. A lower $\dot{V}{\text{O}}_{{2}}$ (− 1 to − 4.5%) was reported during level running at a set running speed with minimalist shoes compared to traditional shoes [111, 250–252]. Similar RE (measured as cost of running) improvements with minimalist shoes were reported during treadmill graded running (slope: + 8% to − 8%; speed: 10 km h^−1^) [28], and from a specific RE (measured as cost of running) protocol (flat and uphill treadmill evaluations) completed before a short trail running session (18.4 km with an alternation of uphill and downhill sections) [111]. Although the metabolic benefit of wearing minimalist shoes is evident in the non-fatigued condition, additional investigations are needed to verify whether these benefits can be preserved with fatigue in trained and experienced minimalist runners.

Hardin and Hamill [149] investigated the effects of specific footwear features (soft-, mid-, and hard-midsole) during DR. Following a 30-min treadmill DR session (slope: − 12%; speed: 12.2 km h^−1^), no difference was reported between midsole conditions on leg shock and EIMD.

In parallel, additional cushioning provided by maximalist shoes has been claimed by manufacturers to provide shock absorption during running, potentially reducing the impact loading and the risk of injury. However, wearing maximalist shoes might not be capable of reducing the impact loading on a level surface and might even increase the external impact loading during short DR treadmill sessions [253].

In summary, current scientific evidence suggests that running footwear is the least effective in-situ strategy to limit the physiological alterations induced by DR. So far, the “metabolic saving” conferred by minimalist shoes has only been recorded in flat running and on non-fatigued muscles.

## Conclusion

Downhill running (DR) is a whole-body exercise characterised by a high proportion of eccentric-biased muscle actions well known to induce muscle damage (EIMD) including neuromuscular fatigue, and ensuing alterations in exercise performance. These physiological alterations generally persist for several days after exercise with a peak recorded between immediately post and 96-h post exercise, depending on the variable that is monitored. Our review shows that in the majority of studies conducted so far, EIMD was assessed through isometric maximal voluntary contraction, blood CK and DOMS, with DR characteristics (slope, exercise duration, and running speed) acting as the main influencing factors. In previous studies, the median (*Q*_1_; *Q*_3_) slope, exercise duration, and running speed were − 12% (− 15%; − 10%), 40 min (30 min; 45 min) and 11.3 km h^−1^ (9.8 km h^−1^; 12.9 km h^−1^), respectively. The training level, and specifically that being used to train on DR sections, can also greatly influence the magnitude of EIMD and must be considered in future studies. Other factors including age and sex might influence the response to DR, though evidence is lacking. Moreover, our analysis highlights the large variety of protocols using the DR model, which prohibits firm conclusions about the factors most responsible for muscle damage and fatigue in DR. In this regard, there is a need for further high-quality research using a consistent approach in the assessment of muscle damage.

The final aim of this review was to gather scientific evidence on strategies implemented in the field to limit EIMD and thus better adapt to DR. We identified two types of strategies; (1) pre-exercise strategies that mainly consist of prior exposure to DR; (2) in-situ strategies that involve the use of specific sportswear (lower limb compression garments and running shoes), and/or voluntary modifications in stride and foot strike patterns. Current scientific evidence shows that prior exposure to DR is the most effective strategy to limit the magnitude of EIMD as a result of the so called “repeated bout effect”. Amongst in-situ strategies, CGs present the most potential but additional studies are required to confirm these findings and understand the underlying mechanisms. Finally, despite a growing interest in the development of innovative running shoes, current data, albeit limited, do not show any influence on the adaptation to DR.

## Declarations

### Availability of data and material

Not applicable to this manuscript.
